# Naturally occurring resistance mutations to inhibitors of HCV NS5A region and NS5B polymerase in DAA treatment-naïve patients

**DOI:** 10.1186/1743-422X-10-355

**Published:** 2013-12-17

**Authors:** Stefania Paolucci, Loretta Fiorina, Bianca Mariani, Roberto Gulminetti, Stefano Novati, Giorgio Barbarini, Raffaele Bruno, Fausto Baldanti

**Affiliations:** 1 Molecular Virology Unit, Virology and Microbiology Department, Fondazione IRCCS Policlinico San Matteo, 27100 Pavia, Italy; 2 Institute of Infectious Diseases, University of Pavia, 27100 Pavia, Italy; 3 Division of Infectious and Tropical Diseases, Fondazione IRCCS Policlinico San Matteo, 27100 Pavia, Italy

**Keywords:** Hepatitis C virus, HCV baseline resistance, NS5A and NS5B genes, DAA inhibitors

## Abstract

**Background:**

Direct-acting antiviral (DAA) agents target HCV proteins; some of these have already been approved for the treatment of HCV infection, while others are in development. However, selection of DAA-resistant viral variants may hamper treatment. The aim of this study was to illustrate potential natural DAA-resistance mutations in the HCV NS5A and NS5B regions of HCV genotypes 1a and 1b from DAA-naïve patients.

**Methods:**

Direct sequencing of HCV NS5A and NS5B regions was performed in 32 patients infected with HCV genotype 1a and 30 patients infected with HCV genotype 1b; all subjects were naïve to DAAs.

**Results:**

In genotype 1a strains, resistance mutations in NS5A (M28V, L31M and H58P) were observed in 4/32 (12.5%) patients, and resistance mutations in NS5B (V321I, M426L, Y448H, Y452H) were observed in 4/32 (12.5%) patients. In genotype 1b, resistance mutations in NS5A (L28V, L31M, Q54H, Y93H and I280V) were observed in 16/30 (53.3%) patients, while resistance mutations in NS5B (L159F, V321I, C316N, M426L, Y452H, R465G and V499A) were observed in 27/30 (90%) patients.

**Conclusions:**

Mutations conferring DAA resistance were detected in NS5A and NS5B of HCV genotypes 1a and 1b from DAA-naïve patients. Although some mutations confer only a low level of resistance, the presence at baseline of mutated HCV variants should be taken into consideration in the context of DAA therapy.

## Background

Hepatitis C virus (HCV) is classified into six genotypes (1–6) and more than 100 subtypes. The most common genotypes in Western countries are 1a and 1b [1]. Peginterferon/ribavirin (PegIFN/RBV) for the treatment of HCV infection is burdened by adverse reactions in at least 10% of patients [2]. Moreover, a sustained virological response is achieved in only 50% of patients infected with HCV genotype 1 [3]. PegIFN/RBV treatment failure is mainly attributed to its low efficacy against genotypes 1 and 4, but also, to some extent to its side effects [3,4]. Recently developed direct-acting antiviral agents (DAAs) are predicted to have a major impact both in combination with PegIFN/RBV, as well as in IFN-free regimens and telaprevir and boceprevir have now been approved as standard of care treatment [5]. Targets for DAA include HCV NS3 protease, NS5B polymerase and NS5A protein which are essential for virus replication. [6-12].

Nevertheless, the combination of a high HCV replication rate, the low fidelity of HCV polymerase and selective pressures by the immune system and drug treatment lead to the *in vivo* development of viral quasispecies with high sequence diversity among various genotypes and subtypes [13,14] with the potential accumulation of virus variants showing mutations with varying degrees of resistance to DAAs [11-13,15-22], even in the absence of pre-existing drug-exposure [17,23-26]. In particular, natural changes in HCV NS5A and NS5B amino acids (aa) associated with reduced drug susceptibility have been observed in treatment naïve patients [17,27,28].

The aim of this study was to illustrate potential DAA-resistant variants in HCV NS5A and NS5B from DAA-naïve patients infected with genotypes 1a or 1b HCV strains.

## Materials and methods

HCV DAA-naive patients referred to our hospital between 2011 and 2012 were included in this study. A comparable number of sequential patients infected with HCV genotypes 1a or 1b was considered in the analysis. From each patient, serum samples were prospectively collected following approval of the study by the Ethics Committee of the Fondazione IRCCS Policlinico San Matteo (protocol no. 20080009620) and after obtaining written informed consent. HCV genotypes were defined using the Versant HCV Genotype 2.0 Assay LiPA (Siemens Healthcare Diagnostic Inc., Tarrytown, NY USA). NS5A and NS5B sequencing was used to differentiate HCV genotypes 1a and 1b. Data were analyzed with the Blast program (http://blast.ncbi.nlm.nih.gov).

Viral RNA was extracted from serum samples using the automatic Easy Mag extractor (Biomerieux, Lyon, France), and full-length HCV NS5A and NS5B genes were amplified using Superscript III One-step enzyme with Platinum Taq (Invitrogen, Carlisbad, CA, USA) in a nested RT-PCR. Primers used in the RT-PCR and nested PCR, spanning NS5A aa 1 to 406 and NS5B aa 1 to 547, are shown in Table 1. The PCR products in the first PCR round were obtained by using the following conditions: 30′ at 45°C for the reverse transcription followed by 10′ at 94°C, and then 50 cycles at 94°C for 1′, 60°C for 1′ and 68°C for 2′, with an extension at 68°C for 10′ in all reactions. Three microliters from the first PCR reaction were used in the nested PCR with the following conditions: denaturation step at 94°C for 10′ and then 30 cycles at 94°C for 1′, 60°C for 1′ and 72°C for 2′, with an extension at 72°C for 10′in the NS5A gene; and denaturation step at 94°C for 10′ and then 30 cycles at 94°C for 1′, 65°C for 1′ and 72°C for 2′, with an extension at 72°C for 10′ in the NS5B gene.

**Table 1:** Amplification and sequencing primers for HCV NS5A and NS5B in genotypes 1a and 1b

**Gene**	**Primer name**	**Primer sequence**
GT1a* NS5A	HCV-1a NS5A Fwd out	GACATCTGGGACTGGATATGYGA
	HCV-1a NS5A Rew out	GTCCAGGWRTARGACATYGAGCA
	HCV-1a NS5A Fwd inn	GATATGYGAGGTGYTGAGCGA
	HCV-1a NS5A Rew inn	GAGCARCACACGACRTCYTC
	HCV-1a NS5A Rew Seq	AAGGAGTCCARRATCACCAC
	HCV-1a NS5B Fwd out	GGAGCCKGGRGATCCRGATCTYAGC
GT1a* NS5B	HCV-1a NS5B Rew out	GTTGGGGAGGAGGTAGATGCCTA
	HCV-1a NS5B Fwd inn	GAYGTCGTGTGCTGCTCRATGTC
	HCV-1a NS5B Rew inn	GAGACACGCTGTGATAAATGTCTCCC
	HCV-1a NS5B Fwd Seq	TCGTAAGCCAGCTCGYCTCATCG
	HCV-1a NS5B Rew Seq	CCTATTGATTTCACCTGGAGAG
	HCV-1b NS5A Fwd out	GGATYAAYGARGACTGYTCYAC
	HCV-1b NS5A Rew out	GACCARGACCCGTCRCTGAGRT
	HCV-1b NS5A Fwd inn	GGGAYTGGATATGCACGGT
**GT1b**^**§ **^**NS5A**	HCV-1b NS5A Rew inn	GGCATGGAGGARTAYGAC
	HCV-1b NS5A Fwd Seq	ARTTGTCTGCGCCTTCYYTGAAGG
	HCV-1b NS5B Fwd out	CGYTGAGTCRTAYTCCTCCATGC
	HCV-1b NS5B Rew out	GGGCRCGAGACASGCTGTGATA
	HCV-1b NS5B Fwd inn	CTCAGYGACGGGTCYTGGTC
**GT1b**^**§ **^**NS5B**	HCV-1b NS5B Rew inn	GCTGTGATATATGTCTCCCC
	HCV-1b NS5B Fwd Seq2	TGGGRGTHCGYGTRTGCGAG
	HCV-1b NS5B Rew Seq3	AGCATYGTGCAGTCCYGGAGC

*Genotype 1a; ^**§**^Genotype 1b.

Direct sequencing of PCR products was performed using an automatic sequencer (ABI PRISM 3100 genetic analyzer DNA Sequencer, Applied Biosystems, Foster City, CA, USA) and the BigDye Terminator v1.1 Cycle Sequencing kit (Applied Biosystems, Foster City, CA, USA). Sequencing primers used to complete the NS5A and NS5B analyses are shown in Table 1.

Nucleotide sequences were assembled using the Sequencer 5.0 (Gene Codes Corp., Ann Arbor, MI) software program. Nucleotide sequences were aligned with reference sequences of different subtypes. GeneBank accession numbers for NS5A and NS5B reference sequences are AF009606 for HCV genotype 1a, and AY045702 for genotype 1b.

The sequences reported in this study have been submitted to the GenBank database under accession numbers KF667756 - KF667879.

## Results

The clinical and virological characteristics of the patients considered in the study are provided in Table 2. Most patients (26/32, 81%) were naive to PegIFN and RBV, while none had ever been treated with HCV NS3, NS5A or NS5B inhibitors. Ten out of 62 (16.2%) patients were co-infected with HIV and under treatment with highly active antiretroviral therapy (HAART), while 52/62 (83.8) were HCV mono-infected (Table 2).

**Table 2:** Patient characteristics by HCV genotype

	**HCV genotype (no. of patients)**	
**Characteristics**	**1a (n = 32)**	**1b (n = 30)**
**Gender**		
Male	22 (68.7%)	17 (56.6%)
Female	10 (31.2%)	13 (43.3%)
Demographics		
Italian	31 (96.8%)	25 (83.3%)
Others	1 (3.1%)	5 (16.6%)
No. of HIV-1/HCV co- infected patients receiving HAART	8 (25%)	2 (6.6%)
Median HCV viral load (UI/ml) in HCV mono-infected patients	1,614,064 (range 6,743-7,985,320)	991,975 (range 3,470-4,381,000)
Median HCV viral load (UI/ml) in HCV/HIV co-infected patients	2,367,635 (range 46,801-7,985,320)	473,597 (range 46,801-900,393)
No. of patients naïve to peg IFN/RBV	26 (81.2%)	29 (96.6%)

HIV, Human Immunodeficiency Virus; HCV, Hepatitis C Virus; HAART, Highly Active Anti-Retroviral Therapy; IFN, Interferon; RBV, Ribavirin.

DAA resistance mutations were detected in both genotypes 1a and 1b strains from DAA naïve HCV patients (Table 3 and Table 4). Overall, the median frequency of single mutations was 4.75% (range 3.1% and 36.6%). In detail, in genotype 1a strains, mutations in the NS5A region associated with resistance to Daclatasvir, Ledipasvir and GSK805 were observed in 4/32 (12.5%) patients (Table 3); mutations in the NS5B region associated with resistance to PSI-352938, Filibuvir and Tegobuvir were observed in 4/32 (12.5%) patients (Table 4). In genotype 1b strains, mutations in the NS5A region associated with resistance to Daclatasvir, Samatasvir, Ledipasvir and GSK805 were observed in 16/30 (53.3%) patients (Table 3); mutations in the NS5B region associated with resistance to Sofosbuvir, Mericitabine, PSI-352938, Tegobuvir, HCV-796, Filibuvir, JTK-109 and Deleobuvir were observed in 27/30 (90%) patients (Table 4). Of note, the mutation V499A was constitutively present in NS5B of all genotype 1a strains and mutation Q30R was present in NS5A of all genotype 1b strains. Overall, HCV NS5A and NS5B resistance mutations in HCV mono-infected patients were found in 30/62 (48.3%) patients.

**Table 3:** Amino acid mutations in the HCV NS5A protein in DAA-naïve patients infected with HCV genotypes 1a (n = 32) or 1b (n = 30)

**DAAs**	**NS5A mutations in DAA-naïve patients**				
	**Genotype 1a**	**Fold change in EC50**	**Genotype 1b**	**Fold change in EC50**	**References**
Daclatasvir	*M28V (1/32, 3.1%)*^α^	1.3	*L28M/V (1/30,3.3%)*	2.0	[15,17,19]
	Q30E/H/R/K		R30H		
Daclatasvir	*L31M/(1/32, 3.1%)*	341	*L31M (2/30, 6.6%)*	3	[15,17,19]
Ledipasvir	*L31M/(1/32, 3.1%)*	140	L31M		[29]
GSK805	*L31M/(1/32, 3.1%)*	>150	L31M		[30]
Samatasvir	L31M		*L31M (2/30, 6.6%)*	3.6	[31]
	P32L		P32L		
Daclatasvir	Q54H/L/N		*Q54H (8/30, 26.6%)*	1.0	[19]
Daclatasvir	*H58P (2/32, 6.2%)*	1.2	P58S/T/L		[19]
	N69T		N69T		
	A92V		A92V		
Daclatasvir	Y93C/H/N		*Y93H (3/30, 10%)*	24	[15,17,19]
Ledipasvir	Y93C/H/N		*Y93H (3/30, 10%)*	1319	[29]
Samatasvir	Y93C/H/N		*Y93H (3/30, 10%)*	93	[31]
	V153M		V153M		
	R157W		R157W		
	V198A		V198A		
	M202L		M202L		
	P223S		P223S		
	M265V		M265V		
GSK805	I280V		*I280V (2/30, 6.6%)*	<2.0	[30]
	V298A		V298A		
	V362A		V362A		
	S364P		S364P		
	S368P		S368P		

^α^Amino acid changes conferring resistance to NS5A inhibitors are reported in italics. The number and % of patients with mutated strains is reported in brackets.

GeneBank accession number of NS5A reference sequence for HCV genotype 1a is AF009606, and for genotype 1b, AY045702.

**Table 4:** Amino acid mutations in the HCV NS5B protein in DAA-naïve patients infected with HCV genotypes 1a (n = 32) or 1b (n = 30)

	**NS5B mutations in DAA-naïve patients**				
**DAAs**	**Genotype 1a**	**Fold change in EC50**	**Genotype 1b**	**Fold change in EC50**	**References**
	S96T^γ^		S96T		
	N142T		N142T		
Sofosbuvir + Mericitabine	L159F		*L159F (7/30, 23.3%)*	X^β^	[32]
	C223H/Y		C223H/Y		
	S282T		S282T		
	L320F		L320F		
PSI-352938	*V321I (1/32, 3.1%)*^*α*^	2.0	*V321I (1/30, 3.3%)*	2.0	[17,33]
Tegobuvir + HCV796	C316Y/N/F/S		*C316N (11/30, 36.6%)*	3.0-5.2	[17,34]
	V362A		V362A		
	S365A/T		S365A/T		
	M414T		M414T		
	L419M/S		L419M/S		
	A421V		A421V		
	R422K		R422K		
	M423I/V/T		M423I/V/T		
Filibuvir	*M426L (1/32, 3.1%)*	0.8	*M426L (2/30, 6.6%)*	0.8	[21]
	C445F		C445F		
Tegobuvir	*Y448H (1/32, 3.1%)*	36.0	Y448H		[34]
Tegobuvir	*Y452H (1/32, 3.1%)*	6.9	*Y452H (1/30, 3.3%)*	6.9	[34]
Tegobuvir	R465G		*R465G (1/30, 3.3%)*	1.1	[34]
	I482L/T		I482L/T		
	A486V		A486V		
	V494A		V494A		
	P495A/L/S/T		P495A/L/S/T		
	P496A/S		P496A/S		
JTK-109 + Deleobuvir	A499T		*V499A (4/30, 13.3%)*	3.0	[17,35]
	G554D		G554D		
	S556G/D/N		S556G/D/N		
	D559G/N		D559G/N		

^α^Amino acid changes conferring resistance to NS5B inhibitors are reported in italics. The number and % of patients with mutated strains is reported in brackets. ^β^Resistance levels are reported only when associated with L320F. GeneBank NS5B reference sequence accession number for HCV genotype 1a is AF009606, and for genotype 1b, AY045702.

HCV NS5A and NS5B resistance mutations in HCV/HIV co-infected patients were found in 3/10 (30%) patients with no statistical difference vs mono-infected patients (p = 0.32). In detail, one patient had the M28V mutation in NS5A, one patient had Y448H in NS5B and one patient had H58P + Y452H in NS5A and NS5B, respectively.

Among the 30 patients infected with genotype 1b strains, two (6.6%) had a mixture of virus variants carrying multiple NS5A resistance mutations, while eight (26.6%) exhibited a mixture of strains with multiple NS5B resistance mutations. In detail, in NS5A of two patients carrying genotype 1b, the mixture Q54H + Y93H was observed. In NS5B of eight patients with genotype 1b, eight different mixtures were observed (L159F + V499A; L159F + C316N; L159F + C316N + V499A; L159F + C316N + M426L; C316N + M426L; C316N + V499A; V321I + V499A and C316N + R465G + V499A). In addition, combinations of multiple resistance variants in both the NS5A and NS5B genes of the same HCV strain, were observed in 1/32 (3.1%) patients with HCV genotype 1a and 8/30 (26.6%) patients with HCV genotype 1b. In particular, a patient with genotype 1a infection had the H58P mutation in NS5A and Y452H in NS5B, while in the eight patients carrying genotype 1b, 3 patients had Q54H in NS5A and C316N in NS5B, one patient had L31M in NS5A and C316N in NS5B, one patient had L31M in NS5A and Y452H in NS5B, one patient had Q54H + Y93H in NS5A and C316N + V499A in NS5B, one patient had Q54H in NS5A and L159F in NS5B and one patient had Q54H in NS5A and L159F + C316N in NS5B.

Notably, all aa variants detected in DAA-naïve patients were low-level resistance mutations except for the mutation Y448H found in the NS5B gene of 1/32 (3.1%) HCV genotype 1a strains which confers higher-level resistance to Tegobuvir and Y93H observed in NS5A of 3/30 (10%) HCV genotype 1b strains which confers higher-level resistance to Daclatasvir, Ledipasvir and Samatasvir.

## Discussion

In this study, 62 patients with genotype 1a or 1b HCV strains were evaluated to determine the frequency of HCV DAA-resistant variants in patients naïve to DAA treatment. The identification of baseline resistance mutations to anti-HCV inhibitors is crucial for defining new therapeutic approaches. Relevant natural aa polymorphisms were found in genotypes 1a and 1b. Some major resistance mutations and other mutations conferring low level resistance to NS5A HCV inhibitors (Daclatasvir, Ledipasvir, GSK805 and Samatasvir), as well as nucleosides (Sofosbuvir, Mericitabine, and PSI-352938) and non-nucleosides (Tegobuvir, Filibuvir, HCV-796, JTK-109 and Deleobuvir) were observed. Similarly, resistance mutations to NS5B HCV inhibitors were confirmed in DAA naïve patients with HCV genotypes 1a and 1b [11,12,17,19,25,29-32,35,36]. Among major mutations in the NS5A gene conferring high level resistance to NS5A inhibitors [17,19,30,31], Q30E/H/K and Y93N/C were not observed, while L31M and Y93H were detected in 2 and 3 patients respectively. A major mutation in the NS5B gene S282T which confers high level resistance to polymerase nucleotide inhibitors [8,17,37], was not observed, while Y448H associated with reduced susceptibility to NS5B non-nucleoside polymerase inhibitors was detected in one patient. Resistance mutations were not observed more frequently in HCV/HIV co-infected patients than in HCV mono-infected patients.

Overall, the median frequency of single mutations observed in the NS5A and NS5B genes analyzed was low as reported in other geographical regions [17,27,28]. In addition, the prevalence of patients with resistance mutations in both genes at baseline was lower in HCV genotype 1a than in HCV genotype 1b infected patients. The higher prevalence of mutations in genotype 1b is due to the presence of C316N, which confers low level resistance to Tegobuvir and HCV-796 in most genotype 1b strains. In addition, in HCV genotype 1b infected patients, the frequency of multiple variant combinations was lower in NS5A than in NS5B. Moreover, in HCV genotype 1a infected patients, combinations of multiple resistance mutations in both NS5A and NS5B of single patients were observed with significant frequency in both HCV genotype 1a and 1b infected patients. It should be underlined that while the Q54H + Y93H combination has already been reported [19] to provide moderate resistance to Daclatasvir, the other combinations have never been investigated, and their level of resistance is not known. In general, greater heterogeneity was confirmed in HCV genotype 1b strains [26,27].

Only 3.1% of patients with HCV genotype 1a and 10% with HCV genotype 1b had viral strains with mutations conferring higher-level resistance to Tegobuvir, Daclatasvir, Samatasvir Ledipasvir and GSK805. Although the presence of preexisting single or double mutations might not confer a significant level of resistance or preclude successful treatment as observed in PI treatment [23], baseline resistance should be taken into consideration in the prospect of HCV IFN-free DAA therapy. Of particular interest are patients carrying combinations of multiple resistance mutations in both the NS5A and NS5B genes, which might increase the possibility of failure in patients treated with multiple DAA containing regimens.

Further studies are needed to better evaluate the role of all variants and the influence which they might have in modulating resistance levels or susceptibility to HCV drugs.

The sustained response in most patients, even when carrying DAA baseline resistance, is probably due to the clearance of HCV. Although there is the possibility that baseline resistant variants may result in viral breakthroughs during treatment [20,27,29], the clinical impact of resistance-mutations in DAA-naïve patients and their influence on the ability of the virus to replicate *in vivo* remain unclear [14,17,25]. Thus, for patients receiving DAA interferon free regimens, or those who will receive in the near future only combined classes of HCV inhibitors, the potential role of DAA-resistant variants prior to treatment should be evaluated in all target genes since their clinical relevance could be useful in the management of new HCV therapies.

### Consent

Written informed consent was obtained from patients for publication of the data in this manuscript and any accompanying images. A copy of the written consent is available for review by the Editor-in-Chief of this journal.

## Competing interests

The authors declare that they have no financial or competing interests.

## Authors’ contributions

SP, LF, BM carried out the molecular analysis. RG, SN, GB, RB participated in the patient enrolment. FB critically revised the manuscript and raised funding to supported the study. SP interpreted the data and wrote the paper. All authors read and approved the final manuscript.
